# Development of a Flow Cytometry-Based Method for Rapid Detection of *Escherichia coli* and *Shigella* Spp. Using an Oligonucleotide Probe

**DOI:** 10.1371/journal.pone.0150038

**Published:** 2016-02-25

**Authors:** Yong Xue, Jon G. Wilkes, Ted J. Moskal, Anna J. Williams, Willie M. Cooper, Rajesh Nayak, Fatemeh Rafii, Dan A. Buzatu

**Affiliations:** 1 Division of Systems Biology, National Center for Toxicological Research, U.S. Food and Drug Administration, Jefferson, AR, United States of America; 2 Division of Microbiology, National Center for Toxicological Research, U.S. Food and Drug Administration, Jefferson, AR, United States of America; 3 Life Sciences Consultant, 515 W. Matthews Ave., Jonesboro, AR, United States of America; Robert Koch-Institute, GERMANY

## Abstract

Standard methods to detect *Escherichia coli* contamination in food use the polymerase chain reaction (PCR) and agar culture plates. These methods require multiple incubation steps and take a long time to results. An improved rapid flow-cytometry based detection method was developed, using a fluorescence-labeled oligonucleotide probe specifically binding a16S rRNA sequence. The method positively detected 51 *E*. *coli* isolates as well as 4 *Shigella* species. All 27 non-*E*. *coli* strains tested gave negative results. Comparison of the new genetic assay with a total plate count (TPC) assay and agar plate counting indicated similar sensitivity, agreement between cytometry cell and colony counts. This method can detect a small number of *E*.*coli* cells in the presence of large numbers of other bacteria. This method can be used for rapid, economical, and stable detection of *E*. *coli* and *Shigella* contamination in the food industry and other contexts.

## Introduction

Pathogenic strains of *Escherichia coli* have been reported to be responsible for human food-borne diseases and death [1]. Cattle are major carriers of enterohemorrhagic *E*. *coli* (EHEC) and contamination of beef by EHEC has caused massive recalls and economic loss to the food industry [2, 3]. Also, EHEC contamination has been implicated in foods other than beef, such as cookie dough, unpasteurized apple juice [4], and spinach [5–7]. There were 5 outbreaks of *E*. *coli* O157:H7, the most important EHEC strain, associated with beef products in 29 states reported by the Centers for Disease Control and Prevention from 2006–2011 [8]. Most recently 1.8 million pounds of ground beef were recalled because of *E*. *coli* O157:H7 contamination [9]. Furthermore, additional shigatoxigenic *E*. *coli* (STEC) non-O157 strains have been associated with outbreaks involving sausages, beef, milk, apple cider, lettuce, and ice cream. See [9] for a review. Rapid and sensitive tests capable of detecting both pathogenic and non-pathogenic *E*. *coli* are potentially useful as screens for general contamination. Products producing negative results can be released into the food distribution system while those yielding positive results can be studied more closely to detect pathogens among the general *E*. *coli* or *Shigella* spp. Furthermore, a quantitative generic *E*. *coli*/*Shigella* assay is also potentially useful to assess the efficacy of disinfection and plant cleaning procedures.

To identify *E*. *coli* in foods, USDA and FDA have established standard methods based on conventional culture incubation and PCR [10, 11]. Plate-culture methods have lower detection limits and lower cost, while the PCR method has greater specificity. However, because both methods stipulate enrichment of the target organism to quantifiable levels, they require a long time to results (TTR): 48 to 56 hours [12].

Our group recently developed an integrated flow cytometer based system (RAPID-B) to detect microbial contamination rapidly with high sensitivity [13, 14]. Using fluorescent dye-conjugated antibodies specific to *E*. *coli*, the system is able to provide quantitative results in minutes, or semi-quantitative results in a few hours if very few cells are present in the sample so that a brief enrichment is required.

Flow cytometry employs a photoelectric method for cell counting, which was developed by Moldavan in 1934 and successfully used to detect airborne bacterial spores by Gucker et al. in 1940 [15]. Historically, flow cytometers analyze cells flowing through the center of a channel in a quartz chamber, arrayed in single file, as a laser beam illuminates each cell and signals are recorded as forward scatter and side scatter. If cells are also labeled with a fluorescent dye, emitted fluorescence passes through a series of filters to establish the wavelength of the emission which is then detected by photomultiplier tubes (PMTs). The collection of information from coincident light signals from the various detectors can be used to analyze characteristics such as size, granularity, response to probe, *etc*., for each cell individually. Fluorescence *in situ* hybridization (FISH) is a robust and sensitive molecular method to detect intact, permeabilized bacterial cells using sequence-specific rRNA-targeted fluorescently-labeled oligonucleotide probes [4]. FISH can be coupled with flow cytometry to accomplish rapid and specific detection of bacteria [16, 17].

Although RAPID-B antibody-based assays have advantages like short assay time, high sensitivity, and the ability to perform analysis directly in food and other difficult matrices, there are still more advantages to be gained from the development of a genetic based assay. Development of highly specific antibody reagents for emerging pathogens is costly and time-intensive. By contrast, genetic-based reagents can be developed quickly at much lower cost, reducing barriers for the rapid development of assays for novel pathogens. Specificity toward a target organism may not be achieved with antibodies in every case. For these reasons it is advantageous to develop genetic-based assays that are compatible with this instrumental platform. In this study, we developed a rapid flow cytometric-based detection method for generic *E*. *coli* using an oligonucleotide probe targeting a 16S rRNA sequence selective for the species (Flow-FISH) [18]. For inclusivity/exclusivity testing, 51 *E*. *coli* strains, 4 *Shigella* strains and 23 non-*E*. *coli* strains were analyzed. Optimal hybridization conditions, assay sensitivity, and linearity were also studied.

## Materials and Methods

### Strains and media

*E*. *coli* serotype O157: H7 strain (ATCC 43895) was used to develop an oligonucleotide probe based *E*. *coli* assay and optimize Flow-FISH conditions. This strain expresses *stx1* and *stx2* genes, which encode Shiga-like toxins I and II proteins, respectively. For inclusivity tests, 5 *E*. *coli* strains from our stock, 45 *E*. *coli* isolates from Dr. Rajesh Nayak in the Division of Microbiology, NCTR, and 4 *Shigella* species from Dr. Fatemeh Rafii in the Division of Microbiology, NCTR were studied. *Shigella* spp. are very similar to *E*. *coli* and were regarded as appropriate for inclusivity in a generic *E*. *coli* assay. *E*. *coli* spp. included both pathogenic and non-pathogenic strains; all *Shigella* spp. were pathogenic. Strain names and sources are listed in Table 1. All *E*. *coli* strains were incubated in Trypticase Soy Broth (TSB) liquid medium (Becton Dickinson and Company, Washington, DC, USA) at 37°C. For the exclusivity study, 23 non-*E*. *coli* bacteria from our inventory were tested. Strain information and growth media are listed in Table 2. BHI: Brain heart infusion medium (Becton Dickinson and Company, Washington, DC, USA); NB: Nutrient broth medium (Becton Dickinson and Company, Washington, DC, USA); TSB: Trypticase soy broth medium (Becton Dickinson and Company, Washington, DC, USA).

**Table 1:** Inclusivity panel result.

Organism	Strain/Source	Detected cells	Result of hybridization
*E*. *coli*	ATCC 43888	76965	+
*E*. *coli*	ATCC 35421	84129	+
*E*. *coli*	ATCC 4157	58194	+
*E*. *coli*	ATCC 35218	53094	+
*E*. *coli*	ATCC 11775	72990	+
*E*. *coli*	ATCC 51446	65731	+
*E*. *coli*	Avian	194122	+
*E*. *coli*	Canine	187535	+
*E*. *coli*	Canine	122905	+
*E*. *coli*	Lemur	87285	+
*E*. *coli*	Bovine	101752	+
*E*. *coli*	Equine	108881	+
*E*. *coli*	Ferret	78513	+
*E*. *coli*	Bovine	77660	+
*E*. *coli*	Avian	46326	+
*E*. *coli*	Equine	82070	+
*E*. *coli*	Feline	27856	+
*E*. *coli*	Canine	25296	+
*E*. *coli*	Equine	27318	+
*E*. *coli*	Bovine	25119	+
*E*. *coli*	Bovine	40767	+
*E*. *coli*	Canine	153532	+
*E*. *coli*	Caprine	33980	+
*E*. *coli*	Canine	64695	+
*E*. *coli*	Bovine	155137	+
*E*. *coli*	Canine	130137	+
*E*. *coli*	Chicken	113101	+
*E*. *coli*	Canine	81565	+
*E*. *coli*	Chicken	125021	+
*E*. *coli*	Canine	102032	+
*E*. *coli*	Quail	139283	+
*E*. *coli*	Canine	112575	+
*E*. *coli*	Bovine	128700	+
*E*. *coli*	Bovine	11576	+
*E*. *coli*	Equine	16978	+
*E*. *coli*	Equine	23954	+
*E*. *coli*	Canine	57014	+
*E*. *coli*	Canine	47425	+
*E*. *coli*	Canine	17924	+
*E*. *coli*	Leopard	53547	+
*E*. *coli*	Canine	37519	+
*E*. *coli*	Humans	45267	+
*E*. *coli*	Humans	34892	+
*E*. *coli*	Humans	75944	+
*E*. *coli*	Humans	40540	+
*E*. *coli*	Humans	63323	+
*E*. *coli*	Humans	133479	+
*E*. *coli*	Humans	80980	+
*E*. *coli*	Humans	169752	+
*E*. *coli*	Humans	92031	+
*E*. *coli*	Humans	133193	+
*Shigella sonnei*	ATCC 25931	118644	+
*Shigella flexneri*	ATCC 15931	65544	+
*Shigella boydii*	ATCC 8700	72190	+
*Shigella dysenteria*	ATCC 11835	22742	+

**Table 2:** Exclusivity panel result.

Organism	Strain/Source	Growth medium	Detected cells^1^	Result of hybridization
*Listeria monocytogenes*	ATCC19115	BHI	0	–
*Listeria grayi*	ATCC 19119	BHI	0	–
*Listeria innocua*	ATCC 33090	BHI	0	–
*Salmonella enterica* serovar paratyphi A	ATCC 11511	NB	0	–
*Salmonella enterica* serovar Paratyphi B	ATCC 8759	NB	0	–
*Salmonella enterica* serovar anatum	ATCC 9270	NB	0	–
*Salmonella enterica* serovar newport	ATCC 6962	NB	0	–
*Salmonella enterica* serovar typhi	ATCC 6539	NB	0	–
*Salmonella enterica* serovar choleraesuis	ATCC 10708	NB	0	–
*Salmonella enterica* serovar montevideo	ATCC 8387	NB	0	–
*Staphylococcus aureus*	ATCC 25923	TSB	0	–
*Citrobacter diversus*	KM11012	NB	0	–
*Pseudomonas aeruginosa*	ATCC 9027	NB	0	–
*Enterococcus durans* Collins et al.	ATCC 11576	TSB	0	–
*Enterobacter cloacae* subsp. cloacae (Jordan) Hormaeche and Edwards, subsp. nov.	ATCC 35030	NB	0	–
*Enterobacter aerogenes* Hormaeche and Edwards	ATCC 35028	NB	0	–
*Pseudomonas aeruginosa* (Schroeter) Migula	ATCC 9027	NB	0	–
*Staphylococcus aureus* subsp. aureus Rosenbach	ATCC 6538	TSB	0	–
*Bacillus cereus* Frankland and Frankland	ATCC 14579	NB	0	–
*Staphylococcus capitis* subsp. capitis Kloos and Schleifer	ATCC 35661	TSB	0	–
*Aerococcus viridans* Williams et al.	ATCC 11563	BHI	0	–
*Acinetobacter sp*.	ATCC 19139	NB	0	–

BHI: Brain heart infusion; NB: Nutrient broth; TSB: Trypticase soy broth

^1^ The number of fluorescent events detected were reduced by a threshold determined empirically from multiple experiments.

### Sample preparation

A generic *E*. *coli* oligonucleotide probe ES445 [19] with sequence: 5’ CTT TAC TCC CTT CCT CCC 3’ was produced and Alexa Fluor^®^ 488 was linked to its 5’ end by Integrated DNA Technologies, Inc., Coralville, IA, USA. Bacterial colonies were picked up from agar plates, inoculated into 5 mL liquid media and cultured in an incubator overnight. The next morning 1 mL from each of the cell cultures was centrifuged at 5000×g for 5 min and the supernatants were removed. Cell pellets were washed with 1 mL phosphate buffered saline (PBS, Sigma-Aldrich, St. Louis, MO, USA) and centrifuged again at 5000×g for 5 min. Cell pellets were suspended in 1 mL 10% buffered formalin (Sigma-Aldrich, St. Louis, MO, USA) and fixed at room temperature for 30 min with slow shaking. After centrifugation at 5000×g for 5 min, pellets were washed with 1 mL PBS and centrifuged at 5000×g for 5 min. Pellets could be stored in 1 mL of 50% ethanol/PBS at -20°C for several weeks prior to analysis. Fixed cells were resuspended in 150 μL hybridization buffer (20 mM Tris, pH 7.5, 0.01% SDS, 3 M NaCl) containing 1 μL of 200 ng/μL DNA probe, and incubated at 55°C for 30 min in a Thermomixer (Eppendorf, Hauppauge, NY, USA). Cells were then washed with 1 mL hybridization buffer without probe at 55°C for 45 min with vortexing (1500 rpm). After centrifugation at 5000×g for 5 min, cell pellets were resuspended in 1mL PBS and used for flow cytometer analysis.

### Flow cytometry analysis

The RAPID-B model 9013 flow cytometer from Vivione Biosciences (Vivione Biosciences, LLC, Pine Bluff, AR, USA) was used for the flow-FISH assay. The excitation source is a solid state 20 mW laser (488nm). It has standard cytometry scatter channels but it has been optimized for detection of small cells by virtue of its low angle scatter detector (LS2) and its high angle scatter detector (LS1). Fluorescence emission can be detected and distinguished within wavelength channels using 3 filters: FL1: green channel collecting light with 515–525 nm; FL2: yellow channel collecting light with 575–600 nm; FL3: red channel collecting light with > 615 nm. All light scatters events and emissions are detected by PMTs. Fluorescence signals from all three channels are collected in order to reduce confusing background noise from autofluorescence [20]. The spectral resolution of light scatter events in the instrument is 130 nm, so it is optimal for detecting and distinguishing bacterial cells.

After the FISH hybridization and wash, cell samples were suspended in 1 mL PBS and analyzed by flow cytometry. The *E*. *coli* O157 antibody-based flow cytometry protocol created by our group [12] was modified and used for the genetic probe assay used in this study. Flow cytometry data were further analyzed and exported using FCS Express 4 Flow Cytometry software (De Novo Software, Los Angeles, CA, USA).

### TPC assay

TPC reagent (Vivione Biosciences, LLC, Pine Bluff, AR, USA) containing thiazole orange (TO) and propidium iodide (PI) is used for cell counting. DNA of live cells could be intercalated by TO, a permanent dye, generating green light fluorescence (FL1 channel). PI can only penetrate dead cells whose membranes are compromised; it quenches the TO, and emits red fluorescence (FL3 channel). Thus, a combination of these two dyes provides a rapid method for total cell quantification, as well as discriminating live and dead cells [21].

The TPC assay was used for cell counting in the linearity study as a control and was performed before formalin fixation. For TPC assay, 1 mL cell culture were centrifuged at 5000×g for 5min. Cell pellets were suspended in 1 mL PBS, mixed with 333 μL TPC reagent, and incubated at room temperature with slow shaking for 6 min. The samples were then analyzed by the RAPID-B flow cytometer using the TPC-specific protocol.

## Results

### Flow-FISH analysis of *E*. *coli*

**Fig 1:**
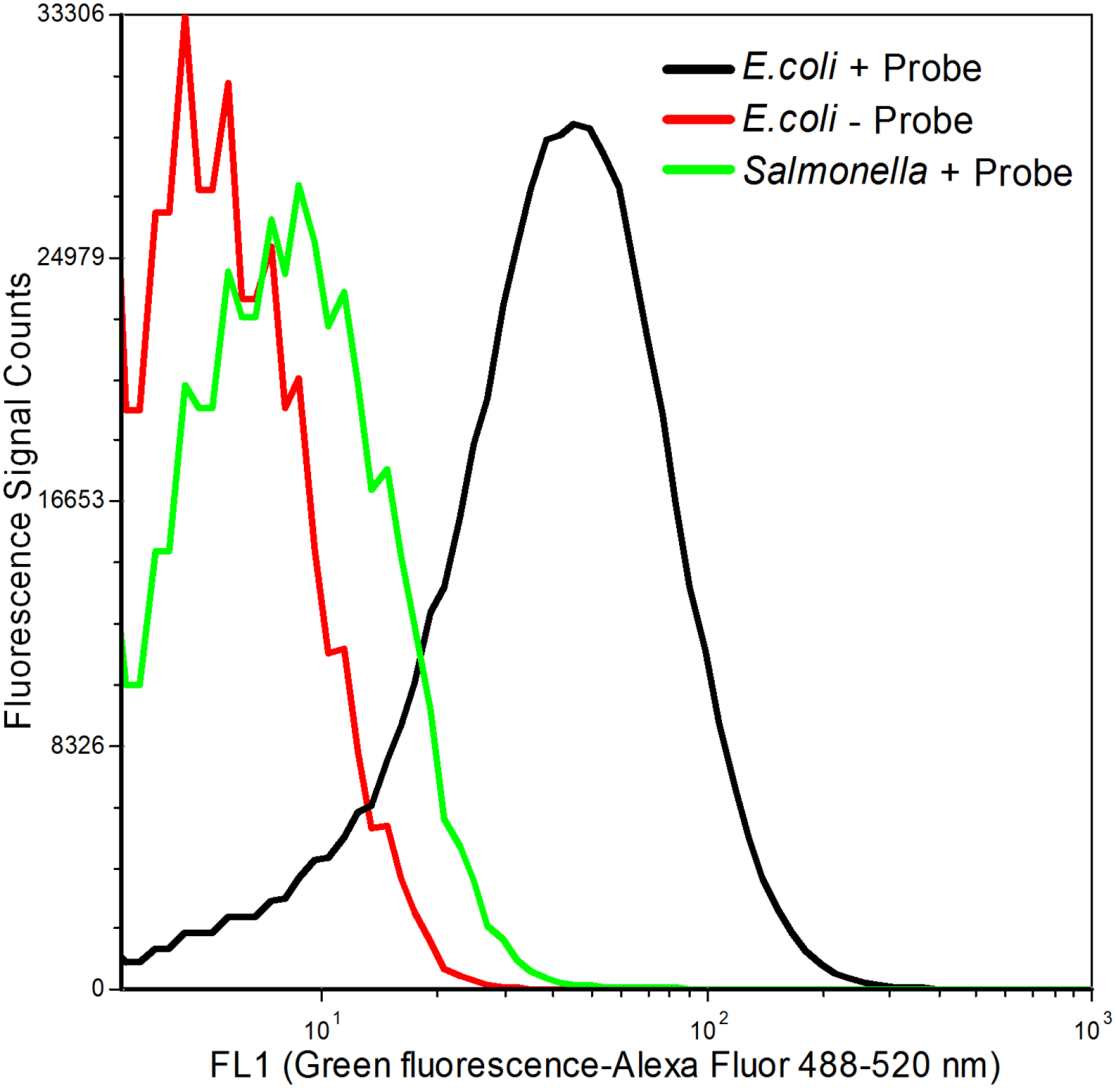
Fluorescence histogram of microorganisms hybridized with probe. Black line: *E*. *coli* O157 labeled with ES445-Alex probe; Red line: *E*. *coli* O157 with no probe; Green line: *Salmonella enterica* serovar choleraesuis (ATCC 10708) labeled with ES445-Alex probe.

Flow cytometry-based detection assay was developed using a 5’-Alexa Fluor 488 dye conjugated-oligonucleotide probe that is specific for 16S rRNA *E*. *coli*. As shown in Fig 1, fluorescently labeled *E*. *coli* cells (black plot) were clearly distinguished from the unlabeled *E*. *coli* cells (red plot), and from *Salmonella* cells (green plot) that were treated with the same fluorochome probe.

Repeating hybridization experiments were performed with the Alexa-Fluor labeled probe in order to optimize detection of *E*. *coli*. The assay was optimized to determine the ideal probe concentration, NaCl concentration in hybridization buffer, hybridization temperature, and time. The optimal hybridization conditions were determined to be: hybridization buffer contains 3 M NaCl and 2 ng/μl DNA probe; hybridization is performed at 55°C for 30 min.

### Inclusivity study

**Fig 2:**
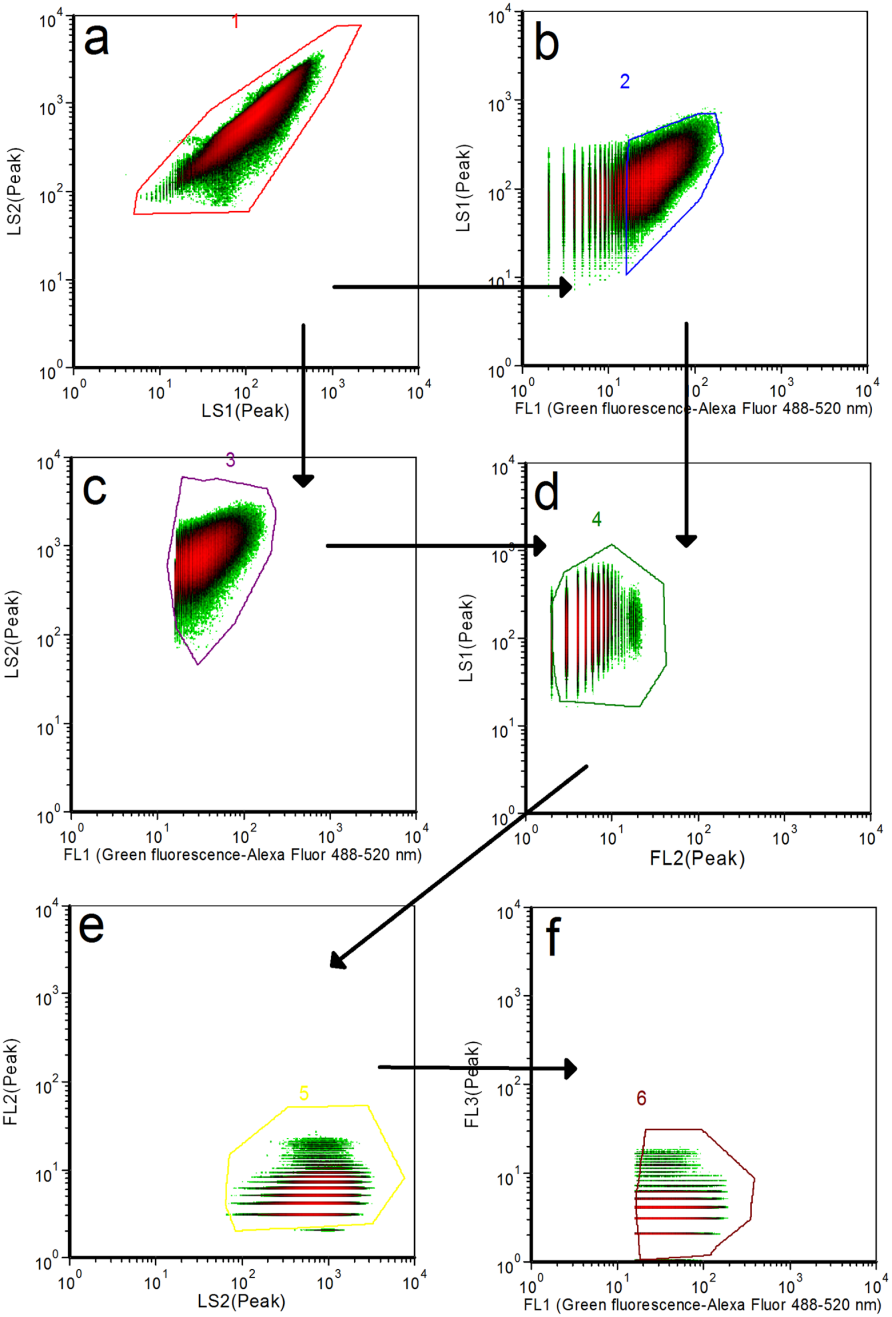
Flow cytometer analysis of *E*. *coli* O157 through serial gates. Signal information from the scatter plot (a) pass through a combination of scatter and fluorescence (b, c, d and e) to the final fluorescence plot (f). Gate logic is indicated by arrows.

**Fig 3:**
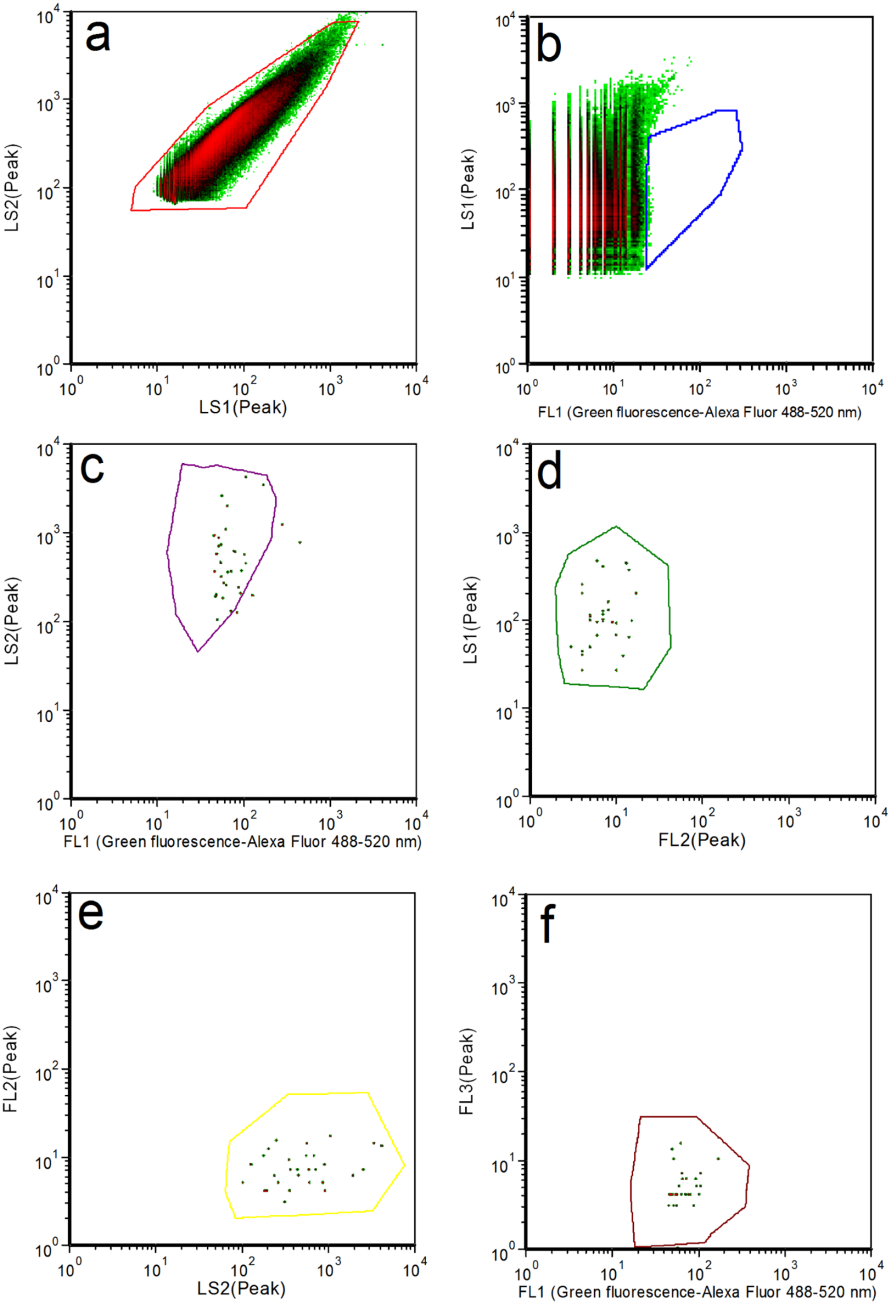
Flow cytometer analysis of *Salmonella enterica* serovar paratyphi A (ATCC 11511) through serial gates. The same gate logic is used as in Fig 2.

After overnight incubation, 1 mL of each *E*. *coli* culture was prepared for inclusivity testing. Flow cytometer assays positively identified all 51 *E*. *coli* strains (Table 1). To get accurate cell counting, a serial gates strategy was developed [12] and used for all inclusivity and exclusivity studies (Figs 2 and 3). Because of the relatively broad emission spectral bandwidth (500–600 nm) of the fluorescent Alexa Fluor 488 labeled probe (Fig 1), the signal of probe-labeled bacteria was present in both FL1 and FL2 channels. The serial gates selection of light scatter parameters and fluorescent intensity includes most target signals and also significantly reduces irrelevant noise. In each plot, a gate indicating the region of interest was determined based on populations (clusters of events with similar combinations of light scatter and emission profiles) from all tested isolates (*E*. *coli* strains and non-*E*. *coli* strains) using FCS Express 4 software.

**Fig 4:**
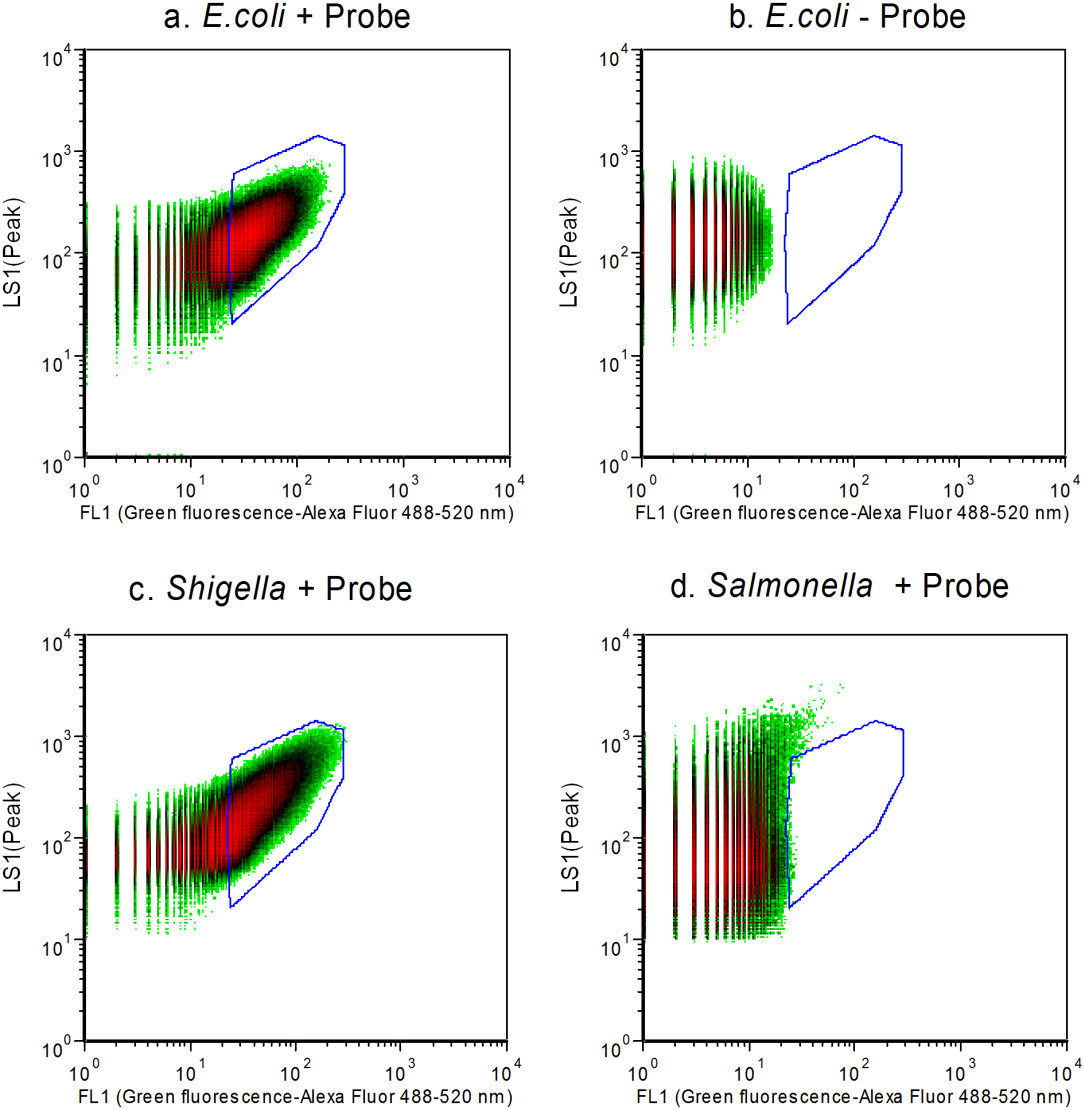
Flow cytometry density-plot of pure cultures hybridized with ES445 probe. LS1: side scatter, FL1: green fluorescence-Alexa Fluor 488–520 nm. A. *E*. *coli* O157 + Probe ES445-Alex; b. *E*. *coli* O157—Probe ES445-Alex; c. *Shigella flexneri* + Probe ES445-Alex; d. *Salmonella enterica* serovar choleraesuis + Probe ES445-Alex.

While testing four *Shigella* strains as part of an exclusivity study, we found that the *E*. *coli* probe could bind all *Shigella* strains and the fluorescence signal appeared within the same set of flow cytometry gates (Fig 4c) as *E*. *coli*. The positive result caused by *Shigella* spp. strains was predicted because of the close genetic relationship between *E*. *coli* and *Shigella* [22].

### Exclusivity study

A total of 27 non-*E*. *coli* strains were tested for specificity by flow cytometry. Due to off-target particulate fluorescence background and chemical noise, a threshold was established to exclude background signals. This was accomplished by performing comprehensive comparisons of negative control samples. A threshold of 100 counts was determined for the exclusivity assay. After subtraction of the threshold from reported counts, remaining positive numbers were recorded as “positive” results.

Except for 4 *Shigella* species, the assay gave negative results for all the other microorganisms (Table 2). Fig 4d shows the flow cytometer density plot of a *Salmonella enterica* strain, one of the negative samples. The Alexa Fluor fluorescence intensity of *Salmonella* in FL1 is much lower than that of *E*. *coli* (Fig 4a).

### Linearity study

**Fig 5:**
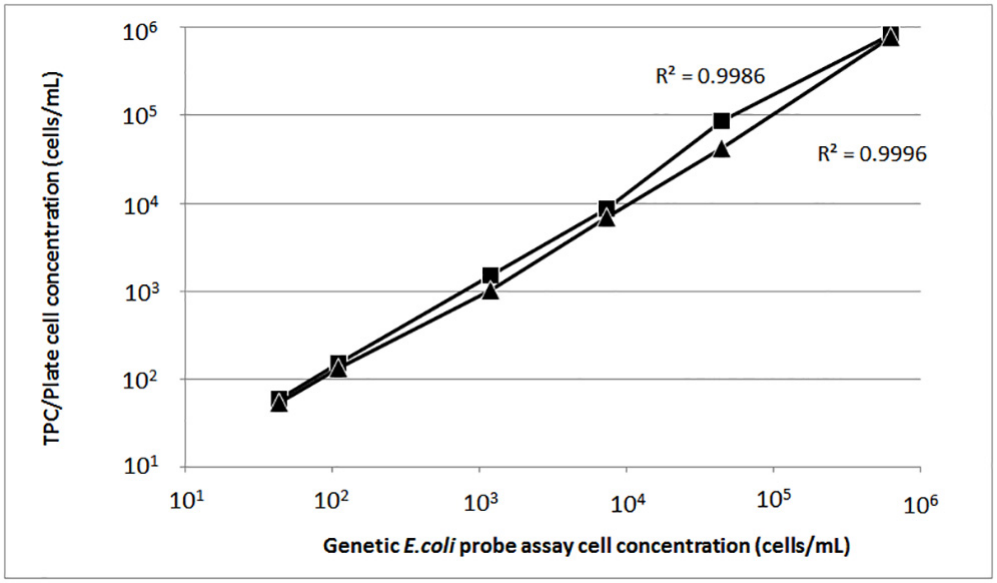
Linearity of generic *E*. *coli* probe assay. Cell culture was diluted over 6 orders of magnitude and equal volume of each dilution were assessed by generic *E*. *coli* probe assay, TPC assay and agar plate counting. The sensitivity of the R-squared value between genetic probe assay and TPC assay (■) is 0.9986, and the R-squared value between genetic probe assay and agar plate counting (▲) is 0.9996. Each data point is an average of 3 repeats.

Serial dilutions of 10^−1^ to 10^−6^ of an *E*. *coli* O157 overnight culture were compared using the genetic *E*. *coli* probe assay, agar plate counting, and the TPC assay. The sensitivity of the R-squared value between genetic probe assay and TPC data is 0.9986, and that between genetic probe assay and agar plate counting is 0.9996 (Fig 5). The sensitivity of generic *E*. *coli* probe assay is comparable to that of the TPC assay and agar plate counting for both low cell density (10^5^−10^6^ cells/mL) and high cell density (10−10^4^ cells/mL).

### Challenge tests

**Fig 6:**
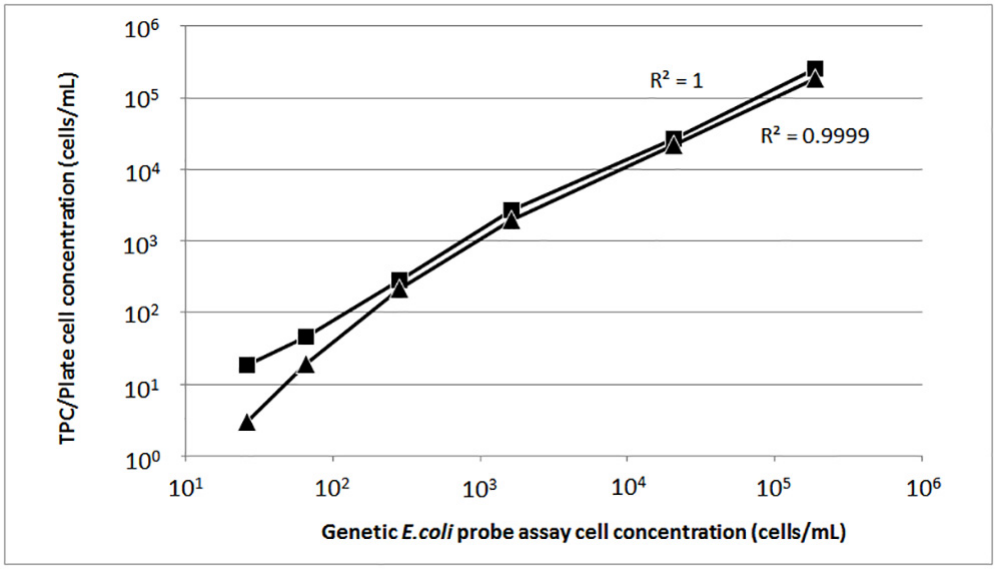
Challenge test of generic *E*. *coli* probe assay. *E*. *coli* O157 culture was diluted over 6 orders of magnitude, mixed with equal volume of *Salmonella enterica* overnight culture, and assessed by generic *E*. *coli* probe assay, TPC assay and agar plate counting. The sensitivity of the R-squared value between genetic probe assay and TPC assay (■) is 1, and the R-squared value between genetic probe assay and agar plate counting (▲) is 0.9999. Each data point is an average of 3 repeats.

We performed challenge tests to determine whether the genetic *E*. *coli* probe assay can distinguish *E*. *coli* from other bacteria, because multiple microbial populations are commonly present in food such as fermented meat and dairy products. *E*. *coli* O157 overnight culture was diluted to 10^−1^ to 10^−6^ and each dilution was mixed with high density of *Salmonella enterica* serovar Choleraesuis (~3×10^6^ cells/mL). As shown in Fig 6, as few as 30–40 cells/mL *E*. *coli* cells can be detected by the genetic *E*. *coli* probe assay in the presence of large number of *Salmonella* cells.

## Discussion

Our study demonstrates that a RAPID-B based method using an oligonucleotide probe can be used for rapid detection and quantification of *E*. *coli* and *Shigella* species. It has potential application in quality control in food industry, environmental, and clinical contexts. It serves as a model for similar RAPID-B assays based on genetic sequences for which the target (here 16S rRNA) has multiple copies present in each cell. This method was tested to have good specificity (exclusivity study) and sensitivity (challenge study). A small number of *E*. *coli* can be detected in the presence of a large number of other bacteria (*Salmonella* is tested here).

Bacterial cells were fixed by formalin treatment and permeabilized to improve accessibility for the oligonucleotides. After the fluorochrome-tagged probe enters permeabilized cells and binds to the target region of 16S rRNA, it can be detected by its green fluorescent emission signal in the FL1 channel. Fixing the cells means that cell viability cannot be determined, unlike the case for antibody-based RAPID-B tests. Also, the sample preparation required for genetic-based detection is more involved and takes more time than detection using antibodies (2 h versus 5 min). However, the resulting fluorescence-tagged cells are stable and therefore more amenable to automated or batch analysis, which reduces TTR and labor when 20 or more analyses are done.

In the study, *Shigella* strains were positively detected by *E*. *coli* generic probe, which is due to the close relationship between *Shigella* and *E*. *coli*. Although *Shigella* was classified as a separate genus according to their medical significance in the 1940s [23], it is more appropriately treated as *E*. *coli* in the phylogenetic perspective [22]. The binding of *E*. *coli* generic probe with *Shigella* isolates was also verified by NCBI BLAST search. 100% identical sequences were found in *Shigella* 16S rRNA.

Although sample preparation takes approximately 2 h for the RAPID-B genetic based assays (versus a few minutes for the antibody-based assays), many samples can be processed in parallel during the same time frame. For example, our laboratory currently has the capability to process up to 24 genetic assay samples in a 2 h period using one Eppendorf Thermomixer R module. Additional centrifuges and Thermomixers would allow even more samples to be prepared in parallel over the same 2 h period. Because of this feature, RAPID-B genetic based assays can be used in food production and safety/regulatory contexts to analyze many samples in meaningful time frames. We are currently developing RAPID-B genetic-based methods for other pathogens including *Listeria monocytogenes*, an organism that is notoriously difficult to label with antibodies.

## Disclaimer

The views presented in this article do not necessarily reflect those of the U.S. Food and Drug Administration.
